# Sulfated flavanones and dihydroflavonols from willow

**DOI:** 10.1016/j.phytol.2019.11.008

**Published:** 2020-02

**Authors:** Clarice Noleto-Dias, Claudia Harflett, Michael H. Beale, Jane L. Ward

**Affiliations:** Department of Computational and Analytical Sciences, Rothamsted Research, West Common, Harpenden, Hertfordshire, AL5 2JQ, UK

**Keywords:** *S.* × *alberti* L. (*S. integra* Thunb. × *S. suchowensis*) hybrid, Salicaceae, Willow, Sulfated flavanones, Sulfated dihydroflavonols, Antioxidant

## Abstract

Phytochemical profiling of a hybrid species of willow, *Salix* × *alberti* L. (*S. integra* Thunb. × *Salix suchowensis* W.C. Cheng ex G.Zhu) revealed four sulfated flavonoids, which were then isolated from young stem tissue. The structures of dihydroflavonols (flavanonols) taxifolin-7-sulfate **(1)** and dihydrokaempferol-7-sulfate **(2)** and flavanones, eridictyol-7-sulfate **(3)** and naringenin-7-sulfate **(4)** were elucidated through NMR spectroscopy and high-resolution mass spectrometry. The identified sulfated flavanones and dihydroflavonols have not been previously seen in plants, but the former have been partially characterised as metabolites in mammalian metabolism of dietary flavonoids. In addition to providing full spectroscopic characterisation of these metabolites for the first time, we also compared the *in vitro* antioxidant properties, *via* the DPPH radical scavenging assay, of the parent and sulfated flavanones, which showed that 7-sulfation of taxifolin and eriodictyol attenuates but does not remove anti-oxidant activity.

## Highlights

• First report in planta of four sulfated flavonoids.
• First report of sulfated flavonoids in the Salicaceae family.
• Sulfated flavanones and dihydroflavonols are rare *in planta*.
• Isolated compounds detected in both leaves and stems at different levels.
• The sulfated form showed less antioxidant capacity compared to non-sulfated flavonoids.

## Graphical abstract

**Figure:**
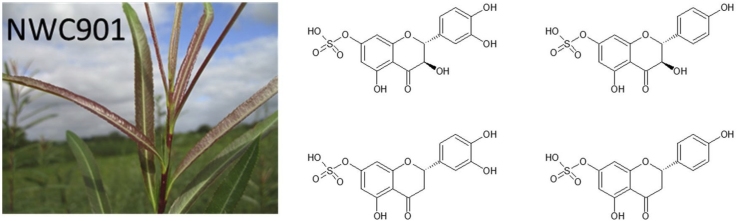

## 1 Introduction

*Salix* L. is the largest genus of Salicaceae family comprising ca. 300–450 species. These deciduous tree and shrub species are generally native to the cooler regions of the Northern Hemisphere, but they have been introduced worldwide (Julkunen-Tiitto and Virjamo, 2016; Lauron-Moreau et al., 2015), due to their useful features, such as fast growth and pleasing appearance, as well as considerable value as medicines and wood products, including biomass for energy (Isebrands and Richardson, 2014). Salicinoids are signature metabolites for Salicaceae family (Boeckler et al., 2011), and are well-known for their role in the development of aspirin (Desborough and Keeling, 2017). However, many other phenolic glycosides (*e.g.* salidroside, triandrin and picein) are also abundant in *Salix* sp., along with several other classes of common plant secondary metabolites, such as cinnamic acid derivatives, condensed tannins, proanthocyanidins and flavonoids (Nyman and Julkunen-Tiitto, 2005; Wiesneth et al., 2018). In our continuing studies on novel phytochemistry in the Salicaceae, in particular those genotypes contained in the 1500+ National Willow Collection (NWC), maintained as a short-rotation coppice plantation at Rothamsted Research, we recently reported on the occurrence of salicin-7-sulfate, a new salicinoid of potential pharmacological interest (Noleto-Dias et al., 2018). Here, we extend the study of sulfated metabolites in *Salix* and now report the isolation, structure determination and radical scavenging activity of four new sulfated flavonoids from the polar extract of a willow hybrid *S.* × *alberti* L. (*S. integra* Thunb. × *S. suchowensis* W.C. Cheng ex G.Zhu).

## 2 Results and discussion

### 2.1 Sulfated flavonoids in a *Salix* hybrid, detected by high mass accuracy tandem mass spectroscopy

**Fig. 1:**
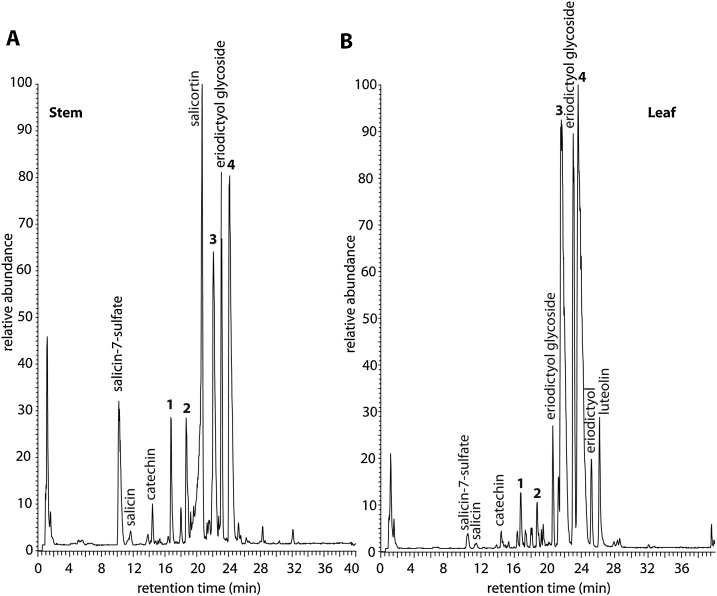
UHPLC total ion chromatograms of stem (A) and leaf (B) tissue of *S.* × *alberti* L. (*S. integra* Thunb. × *S. suchowensis* W.C. Cheng ex G.Zhu) following extraction with H_2_O:MeOH (4:1). Numbers 1–4 correspond to compounds described in the results section and in Fig. 2.

Interspecific hybridisation in *Salix* is frequent, and many of the accessions in the NWC are hybrids sourced from around the globe. These consist of both natural hybrids and those which are generated in breeding programmes for research or improvement of specific traits. NWC901, a hybrid representing *S.* × *alberti* L. (*S. integra* Thunb. × *S. suchowensis* W.C. Cheng ex G.Zhu), is one such example and is a line previously used in the Rothamsted willow breeding programme as a parent for the generation of biparental “mapping population F” (Hanley and Karp, 2014). In our LC–MS metabolomics screens NWC901 stood out as being atypical compared to other willow species, particularly in its flavonoid composition (Nyman and Julkunen-Tiitto, 2005). Stems and leaves of rapidly growing plants were extracted with water:methanol (4:1) and the extracts analysed by UHPLC-MS. As well as the presence of significant levels of salicin-7-sulfate (Noleto-Dias et al., 2018) in the stem samples, the total ion chromatograms (Fig. 1) presented four peaks, present in both leaf and stem samples, with characteristics of sulfated flavonoids, *i.e.* more polar than the corresponding parent flavonoid (see leaf chromatogram, Fig. 1B), and molecular ions corresponding in accurate mass and formulae, to flavonoids bearing a sulfate (−OSO_3_H) as well as major MS fragments corresponding to the loss of SO_3_ (80 a.m.u). In addition, the high mass resolution and accuracy of the applied MS method supported the presence of a sulfur atom in the compound structures as their mass spectra showed [(M−H) + 2]^−^ ions containing the ^34^S isotope, that were clearly distinguishable from the ^18^O isotopomer of very similar mass (Figs. S1 and S2). Tandem mass spectrometry (MS–MS) indicated that peaks 1 and 2 could be classified as (dihydro)flavonols and peaks 3 and 4 were flavanones, by virtue of the fragmentation patterns formed by successive losses of H_2_O, CO, CO_2_ and C_3_O_2_ as demonstrated by Schmidt through analysis of negative ion ESI MS-MS data of a wide range of flavonoids (Schmidt, 2016).

### 2.2 Isolation and structural characterisation

NMR is known to be a good tool to determine the position of sulfate substitution in benzenoid molecules, due to predictable changes in both carbon and proton chemical shifts of atoms attached to, and neighbouring, the sulfate group when compared to those of their respective “non-sulfated” free hydroxy group analogues. In summary, the effect of sulfation of a phenolic hydroxyl group is an upfield shift by *ca.* 4 ppm of the attached carbon, due to an increase in electron density. On the other hand, the *ortho* effect of sulfation causes downfield shifts of 3–5 and *ca.* 0.45 ppm of carbon and proton chemical shifts, respectively, due to a lower electron density (Enerstvedt et al., 2017; Ibrahim, 2000; Op de Beck et al., 1998; Ragan, 1978). Therefore, to obtain clear NMR spectra of each compound and to unambiguously establish the position of the sulfate group, and to confirm the flavonoid parent structure, a larger amount of stem tissue was extracted and subjected to preparative HPLC for compound isolation and structural characterisation.

**Fig. 2:**
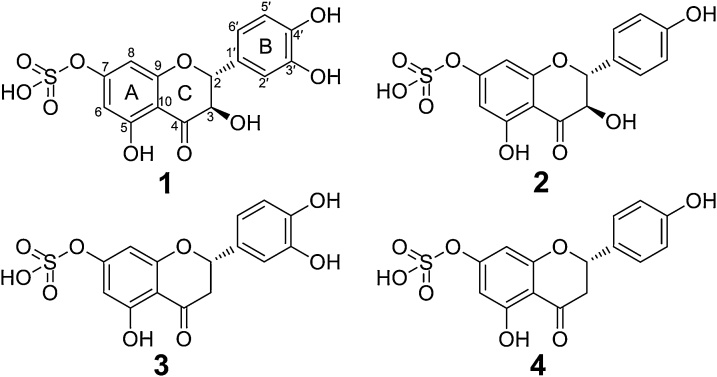
Structures of sulfated flavonoids present in aerial tissue of *S.* × *alberti* L. (*S. integra* Thunb. × *S. suchowensis* W.C. Cheng ex G.Zhu). **1**: taxifolin-7-sulfate; **2**: dihydrokaempferol-7-sulfate; **3**: eriodictyol-7-sulfate; **4**: naringenin-7-sulfate.

Compound **1** (Fig. 1, Fig. 2) corresponded to the peak appearing at 16.8 min in the LC—MS chromatogram and was obtained as a yellow amorphous powder. Its molecular formula was assigned as C_15_H_12_O_10_S on the basis of its HR-ESI-MS (pseudo-molecular ion peak [M–H]^−^ at *m/z* 383.0079) (Fig. S1). The MS–MS spectrum (Fig. S3) showed a base peak at *m/z* 303.0528 [M−H−SO_3_H]^−^ and other peaks appeared at *m/z* 285.0425 [M−H–SO_3_–H_2_O]^−^, 275.0574 [M−H–SO_3_−CO]^−^, 259.0623 [M−H–SO_3_−CO_2_]^−^, 241.0518, 177.0204 and 151.0045 and were characteristic fragment ions of taxifolin (Yang et al., 2016). The ^1^H NMR spectrum of isolated compound **1** showed aromatic signals at δ 7.01 (1H, dd, J = 2.0, 8.2), 7.10 (1H, d, J = 2.0), 6.97 (1H, d, J = 8.2 Hz) related to the B-ring (H-2′, H-6′ and H-5′, respectively) (Table 1). The dihydroflavonol C-ring moiety was evident from the ^1^H and ^13^C NMR signals of C-2, [δ_H_ 5.19 (1H, d, J = 12.0) and δ_C_ 86.5], C-3 [δ_H_ 4.86 (1H, d, J = 12.0) and δ_C_ 75.3], and for C-4 δ_C_ 201.6. The coupling constant of 12 Hz confirms the diaxial relationship of H-2 and H-3, and the stereochemical arrangement in ring C, identical to that of a taxifolin reference standard acquired in the same solvent (Table 1, Figs. S4–S7). Compared to the ^1^H NMR data of taxifolin, the chemical shift of both H-6/C-6 (at δ_H_ 6.47 and δ_C_ 103.8) and H-8/C-8 (at δ_H_ 6.54 and δ_C_ 104.9) were downfield shifted by *ca.* 0.48 and 4.6 ppm for proton and carbon atoms, respectively. On the other hand, an upfield shift of 4.1 ppm was observed for the carbon attached to the sulfate group (C-7 at δ_C_ 161.3) and 4.8 and 3.1 ppm for carbons C-5 and C-9, respectively at meta position to C-7 (Table 2). Therefore, compound **1** was characterized as taxifolin-7-sulfate (Fig. 2). It has not been previously reported in plants and consequently comprises a novel sulfated dihydroflavonol. Unspecified taxifolin sulfates have been previously described as products of the metabolism of 7-galloyltaxifolin in an *in vitro* cell model with human hepatocytes (Vacek et al., 2013) and of taxifolin in rats (Yang et al., 2016).

**Table 1:** Chemical shift data of taxifolin-7-sulfate (**1**), dihydrokaempferol-7-sulfate (**2**), eriodictyol-7-sulfate (**3**), naringenin-7-sulfate (**4**).

Position	taxifolin-7-sulfate **(1)**			dihydrokaempferol-7-sulfate **(2)**			eriodictyol-7-sulfate **(3)**			naringenin-7-sulfate **(4)**		
	δ_C_	δ_H_	*J*_H-H_ (Hz); multiplicity	δ_C_	δ_H_	*J*_H-H_ (Hz); multiplicity	δ_C_	δ_H_	*J*_H-H_ (Hz); multiplicity	δ_C_	δ_H_	*J*_H-H_ (Hz); multiplicity
2	86.5	5.19	12.0; *d*	86.4	5.26	12.0; *d*	82.1	5.54	3.2, 12.3; *dd*	82.4	5.58	3.1, 12.5; *dd*
3	75.3	4.86	12.0; *d*	75.2	4.90	12.0; *d*	44.9	2.94	3.2, 17.4; *dd*	45.3	2.93	3.1, 17.4; *dd*
								3.34	overlapped		3.36	12.5, 17.4; *dd*
4	201.6	–	–	201.4	–	–	202.0	–	–	202.3	–	–
5	162.7	–	–	163.1	–	–	165.3	–	–	165.8	–	–
6	103.8	6.47	2.1; *d*	104.9	6.55	2.1; *d*	103.8	6.49	2.2; *d*	103.5	6.49	2.2; *d*
7	161.3	–	–	162.7	–		163.3	–	–	162.7	–	–
8	104.9	6.54	2.1; *d*	103.5	6.46	2.1; *d*	103.8	6.50	2.2; *d*	104.5	6.50	2.2; *d*
9	165.2	–	–	165.3	–	–	162.5	–	–	165.3	–	–
10	107.5	–	–	107.4	–	–	108.5	–	–	108.8	–	–
1′	131.3	–	–	130.8	–	–	133.2	–	–	132.9	–	–
2′	118.8	7.11	2.0; *d*	133.2	7.49	8.6; *d*	117.6	7.06	1.9; *d*	131.8	7.45	8.6; *d*
3′	147.5	–	–	119.0	6.99	8.6; *d*	146.8	–	–	118.8	6.96	8.6; *d*
4′	148.7	–	–	160.0	–	–	147.7	–	–	159.6	–	–
5′	119.3	6.98	8.2; *d*	119.0	6.99	8.6; *d*	119.4	6.95	8.2; *d*	118.8	6.96	8.6; *d*
6′	124.4	7.02	2.0, 8.2; *dd*	133.2	7.49	8.6; *d*	122.4	6.97	1.9, 8.3; *dd*	131.8	7.45	8.6; *d*

Data collected in D_2_O:CD_3_OD (4:1) at 600 MHz for ^1^H analysis and 150 MHz for ^13^C analyses. Spectra were referenced to d_4_-TSP at δ 0.00. *d* doublet; *dd* double doublet.

**Table 2:** Relative changes in the ^1^H and ^13^C NMR chemical shifts, expressed in ppm as δ (sulfate flavonoid) – δ (flavonoid).

Position	Taxifolin-7-*O*-sulfate **(1)**		Eriodictyol-7-*O*-sulfate **(3)**		Narigenin-7-*O*-sulfate **(4)**	
	δ_C_	δ_H_	δ_C_	δ_H_	δ_C_	δ_H_
*ipso* (7)	–4.1	–	–4.2	–	–4.2	–
*ortho* (6)	+4.6	0.49	+5.0	+0.47	+4.6	+0.45
*ortho* (8)	+4.6	0.48	+5.0	+0.46	+4.9	+0.45
*meta* (5)	–4.8	–	–5.3	–	–4.8	–
*meta* (9)	–3.1	–	–2.4	–	–3.1	–
*para* (10)	+3.8	–	+3.8	–	+3.8	–

Compound **2** eluted at 18.6 min in the chromatographic run and exhibited a [M−H]^−^ at *m/z* 367.0130, with a predicted molecular formula of C_15_H_12_O_9_S, confirmed by the presence of the expected ^34^S isotope ion (Fig. S1). The MS-MS spectrum had *m/z* at 287.0660 [M−H–SO_3_H]^−^ as the base peak and other fragments at *m/z* 259.0610 [M−H–SO_3_−CO]^−^, 243.0661 [M–H–SO_3_−CO_2_]^−^, 219.0666 [M−H–SO_3_–C_3_O_2_]^−^, 201.0555, 180.0066 and 125.0246 that are all consistent with a derivative of the flavonoid dihydrokaempferol (Fig. S8). Compound **2** was isolated as a yellow amorphous powder and its ^1^H NMR spectrum (Table 1) confirmed the B-ring structure by signals of a *para*-disubstituted benzene appearing as doublets of an A_2_B_2_ spin system at δ 7.49 (2H, J = 8.6, H2′/H-6′) and 6.99 ppm (2H, J = 8.6, H3′/H-5′). Furthermore, two oxymethines for the C ring at δ 5.26 (1H, d, J = 12.0, H-2) and 4.90 ppm (1H, d, J = 12.0, H-3) and two *meta*-coupled aromatic protons at 6.55 (1H, d, J = 2.1, H-6) and 6.46 ppm (1H, d, J = 2.1, H-8) for the A ring confirmed the remainder of the dihydrokaempferol skeleton (Figs. S9–S12). A standard of dihydrokaempferol itself was not available for comparison, but for the A-ring chemical shifts, taxifolin was used as a reference and δ_H_ and δ_C_ of the atoms at position 6 were downfield shifted by 0.46 and 5.0 ppm, respectively. Therefore, given the similarity of chemical shifts of rings A and C atoms between compound **1** and **2**, and the relative changes in chemical shifts on sulfation, compound **2** was identified as dihydrokaempferol-7-sulfate (Fig. 2). This compound has been previously suggested as a taxifolin metabolite in rats (Yang et al., 2016), but no NMR data was presented in that study.

Compound **3**, with the same molecular formula (C_15_H_12_O_9_S) as compound **2,** eluted at 22.2 min with an *m/z* of 367.0131. Although its MS–MS spectrum (Fig. S13) showed an identical base peak (at *m/z* 287.0563), the other fragments were different, at *m/z* 269.0465 [M−H–SO_3_–H_2_O]^−^, 177.0202, 161.0243, 151.0038, 135.0453 and 125.0246, indicating a different base structure. In addition to these peaks, a fragment at *m/z* 230.9628 (C_7_H_4_O_7_S) is indicative of the presence of the sulfate group attached to the A ring. Compound **3** was isolated as a yellow amorphous powder and in the ^1^H NMR (Table 1), presented aromatic signals of an ABX spin system at δ 7.06 (1H, d, J = 1.9, H-2′), 6.97 (1H, dd, J = 1.9, 8.3, H = 6′) and 6.95 ppm (1H, d, J = 8.2, H-5′) corresponding to protons in the flavonoid B ring and δ 6.50 (1H, d, J = 2.2, H-6) and 6.49 (1H, d, J = 2.2, H-8) for those residing in the A ring. Other proton signals corresponded to one oxymethine at δ 5.54 ppm (1H, dd, J = 3.2, 12.3, H-2) and two methylenes at δ 3.34 (overlapped, H-3) and 2.94 ppm (1H, dd J = 3.2, 17.4, H-3) for those protons in the C ring (Figs. S14–S17). Its ^1^H NMR spectrum resembled that of authentic eriodictyol obtained under the same conditions, however the chemical shifts of H-6 and H-8 were shifted downfield at 0.46 ppm by the *ortho* effect of the sulfate group at C-7 (Table 2). Based on this evidence, compound **3** was characterized as eriodictyol-7-sulfate (Fig. 2). This compound had been previously suggested, on the basis of MS data alone, as a metabolite of dietary taxifolin in rats (Yang et al., 2016). However, as for **1** and **2**, this is the first report of the occurrence of this compound as a natural product in plants, and the first time the structure has been elucidated by NMR.

Compound **4** had a retention time of 24.2 min and was purified as a colourless amorphous powder. Its molecular formula was assigned as C_15_H_12_O_8_S on the basis of its high resolution ESI-MS ([M−H]^−^ at *m/z* 351.0183) (Fig. S2). ^1^H and ^13^C NMR data of the isolated compound (Table 1, Fig. S18–S22) were consistent with the described data of naringenin-7-sulfate (**4**) (Fig. 2), a compound previously fully characterised as a product of naringenin bio-transformation by the fungus *Cunninghamella elegans* (Ibrahim, 2000). To our knowledge this compound has not been reported as a plant natural product before.

The presence of sulfated flavonoids has been shown for the first time in the genus *Salix*. This family of around 150 compounds has been previously described in a significant number of Angiosperm species, especially in the Asteraceae family. However, the vast majority of sulfated flavonoids are from flavonol and flavone subclasses (Barron et al., 1988; Correia-da-Silva et al., 2014; Teles et al., 2018). Only one sulfated flavanone (naringenin-4′-sulfate) and one sulfated dihydroflavonol (dihydromyricetin-3′-sulfate) have previously been recorded in any plants (Teles et al., 2018). Sulfation at C-7 is fairly common. The physiological role of sulfated flavonoids is unclear, although in aquatic plants such as the seagrass *Zostera noltei* Hornem. (Zosteraceae) the appearence of these compounds has been suggested to be related to the high concentration of sulfate ion in seawater (Grignon-Dubois and Rezzonico, 2018).

### 2.3 Antioxidant activity of sulfated flavonoids

As bioactive products, sulfated flavonoids have the advantage of being more water-soluble, which is an interesting property for clinical preparations (Correia-da-Silva et al., 2014). Some bio-activities have been described for sulfated flavonols, for example, sodium morin-7-sulfate exhibited anticancer properties against melanoma cells (Li et al., 2016); myricetin-3′-sulfate was active against *Trypanosoma brucei* with IC_50_ value of 8.52 μg/ml. However, this and two additional sulfated flavonoids isolated from *Limonium caspium* Gams (Plumbaginaceae) did not show antifungal activity against *Candida glabrata* or antimalarial activity (Gadetskaya et al., 2015). Quercetin tri- and tetra-sulfates isolated from *Flaveria bidentis* (L.) Kuntze (Asteraceae) showed significant anticoagulant effects *in vitro*, probably due to their high charge density properties (Guglielmone et al., 2002).

Flavonoids are considered antioxidants and the radical scavenging activities of the isolated compounds were assessed by the well-known DPPH-test in order to evaluate the effect of the sulfate moiety on this activity compared with the non-sulfated flavonoids. The results showed that taxifolin-7-sulfate (**1**) and eriodictyol-7-sulfate (**3**) were over 3-fold less active than taxifolin and eriodictyol (Table 3), respectively, whilst dihydrokaempferol-7-sulfate (**2**), naringenin and naringenin-7-sulfate (**4**) were inactive (IC_50_ > 500 μM). Much greater activity was observed for taxifolin and eriodictyol than for naringenin, underlining the known importance of the B-ring catechol function for high antioxidant activity (Pietta, 2000). The reduction of activity (to *circa* 30 % of the parent) by 7-sulfation, observed here for taxifolin and eriodictyol, is consistent with previous work that shows the reduction of the antioxidant activity by sulfation (Roubalová et al., 2015). However, in that study, the sulfation at the B ring of quercetin and taxifolin (*i.e.* quercetin 3′-sulfate and taxifolin-4′-sulfate) was seen as the reason for this attenuation, while in the present study we show a similar reduction in radical scavenging activity for sulfation at the A ring. Thus, sulfation could reduce bioactivity in the same way that was shown for flavonoid glycosides *versus* their aglycones (Nguyen et al., 2017). However, it is important to realise that in the DPPH assay we are not mimicking the mammalian system and the potential endogenous transport and metabolism of such conjugated flavonoids compounds after ingestion should be kept in mind.

**Table 3:** DPPH radical scavenging activity of flavonoids.

Compound	IC_50_ ± SD (μM)
Taxifolin	48.0 ± 2.6
Taxifolin-7-sulfate (**1**)	159 ± 4.4
Dihydrokaempferol-7-sulfate (**2**)	>500
Eriodictyol	64.1 ± 3.6
Eriodictyol-7-sulfate (**3**)	196.4 ± 11
Naringenin	>500
Naringenin-7-sulfate (**4**)	>500

## 3 Concluding remarks

In this study, four sulfated flavonoids were isolated from the leaves and stems of *S.* × *alberti* L. (*S. integra* Thunb. × *S. suchowensis* W.C. Cheng ex G.Zhu) hybrid and their structures were fully determined by spectroscopic studies. This is the first report of these compounds in a plant material source. It is of note that the majority of sulfated flavonoids found in plants are of the flavone and flavonol types, and sulfated analogues of the flavanone and dihydroflavonol classes are very rare (Teles et al., 2015, 2018; Fernandes et al., 2019). *Salix* species were not known for sulfated metabolites until recently, when salicin-7-sulfate was shown to be relatively common across the genus (Noleto-Dias et al., 2018). The work described here provides evidence that sulfation is a metabolic course that may be more generally distributed in *Salix* secondary metabolism. It is of some significance that the sulfation in the species studied appears to occur at an earlier point than normally observed in the plant flavonoid biosynthesis pathway. Although luteolin (a flavone) was observed in leaf tissue (Fig. 1B) generally the flavones and flavonols are much less abundant in this line. It appears that the sulfation process is a way of removing excess precursor flavanones and dihydroflavonols from a pathway with restricted flow towards the end products. The observed flavonoid sulfation reaction is likely a complement to glycosidation for metabolism of flavonones such as eriodictyol, the glycoside of which is also evident in this line (Fig.1). In this willow genotype sulfation is regio-specific at the 7-OH position. The same specificity was previously observed for the human sulfotransferase isoform SULT1A3 (Meng et al., 2012) and for the isoforms AtSULT202B7 and AtST3a from *Arabidopsis thaliana* (Gidda and Varin, 2006; Hashiguchi et al., 2014). In animals, the sulfotransferases are part of the first-pass metabolism of xenobiotics and endogenous compounds, which is one of the most important phase II reactions for the biotransformation and/or excretion of these metabolites. Here we have demonstrated that, *in vitro*, 7-sulfates retain significant (30 %) antioxidant activity and thus such compounds may still have a bioactive role in mammalian systems if generated *in vivo* from ingested flavonoids. On the other hand, the physiological role in plants, other than a detoxification process, is unclear. It remains to be seen whether the sulfotransferases involved in salicin conjugation and flavanone/dihydroflavonol conjugation are the same and further studies on the identification and characterisation of these enzymes will be facilitated by tracking the origin of the trait back through the parents and progeny of the hybrid utilised in this study, thus supporting a genetic mapping approach to identifying the sulfotransferase genes that may be involved.

## 4 Experimental

### 4.1 General analytical procedures

Acetonitrile, methanol, and formic acid (LC–MS grade) were obtained from Fisher Scientific (Loughborough, UK) and milliQ water was used for extraction and/or HPLC analysis. All other chemicals and standards were purchased from Sigma Aldrich (UK). NMR spectra were acquired on an AVANCE 600 MHz NMR Spectrometer equipped with a 5 mm TCI cryoprobe (Bruker Biospin, Coventry, UK) and experiments were conducted as previously reported (Noleto-Dias et al., 2018). For LC–MS analyses, the data were recorded with an Dionex UltiMate 3000 RS UHPLC system, equipped with a DAD-3000 photodiode array detector (PDA), coupled to an LTQ-Orbitrap Elite mass spectrometer (Thermo Fisher Scientific, Germany), as previously reported (Noleto-Dias et al., 2018). UV spectra were derived from the PDA in the LC–MS runs. Optical rotation was measured in water on an Anton Paar MCP-100 polarimeter using a 100 mm sample cell. Absorbance (UV–vis) was measured in a Varioskan™ LUX multimode microplate reader (Thermo Fisher Scientific, Germany).

### 4.2 Plant growth and preparation

20 cm dormant cuttings of *S.* × *alberti* L. (*S. integra* Thunb. × *S. suchowensis* W.C. Cheng ex G.Zhu) were planted to half depth in 7 L pots measuring 15 cm diameter and 30 cm tall containing a mixture of 50:50 perlite and Rothamsted Standard Compost Mix (75 % peat, 12 % loam, 3 % vermiculite and 10 % lime-free grit with Osmocote Exact 3−4 M added at 3.5 kg/m^3^). A single *Salix* cutting was planted per pot and plants were grown under controlled environment conditions in a Gallenkamp growth room (Sanyo, Loughborough, UK). The growth chamber temperature was 10 °C night/18 °C day. Humidity was set at 60 % and 90 % for day (14 h) and night (10 h) respectively. Average light levels at plant growth height (1.2 m from light source) were 600 μmol m^−2^ s^−1^. Plants were grown for 62 days before harvesting of the top 20 cm portion of the lead stem directly into liquid nitrogen. Tissues were kept at −80 °C prior to freeze-drying to remove residual water. Stems and leaves were separated and milled to fine powders (Retsch Ultra Centrifugal Mill ZM200, Retsch, UK). Milled tissues were maintained at −80 °C until sample extraction. Voucher specimens have been retained and are available on request.

### 4.3 Extraction and isolation of metabolites

Milled freeze-dried stem material (178 mg) was suspended in 9 ml H_2_O:CH_3_OH (4:1 v/v). The extract was vortexed vigorously and heated at 50 °C in a water bath for 10 min and then centrifuged at 13,200 rpm. The supernatant (6 ml) was transferred to a clean tube, which was then heated at 90 °C for 2 min and stored at −4 °C for 30 min. The sample was centrifuged for 10 min at 13,200 rpm and 5 ml was aliquoted and placed in glass autosampler vial. Isolation of compounds was achieved using a HPLC system (Dionex UltiMate 3000, Thermo Fisher Scientific) equipped with an Ascentis C-18 column (5 μm, 5 × 250 mm i.d., Supelco, UK) maintained at 35 °C. The chromatographic separation was performed by using a constant flow rate of 1 ml/min of the mobile phases water (A) and acetonitrile (B), both with 0.1 % formic acid. The binary gradient was: 0–5 min, linear from 5 to 12 % B; 5–70 min, linear from 12 to 40 % B, followed by 10 min wash (100 % B) and 10 min re-equilibration (5 % B). Peaks were detected using wavelengths of 210–360 nm and were automatically collected by time into glass tubes. Forty-one injections (100 μl each) were performed and equivalent fractions from repeated runs were combined and the solvent was evaporated using a Speedvac concentrator (Genevac, Suffolk, UK).

### 4.4 Spectroscopic data

Taxifolin-7-sulfate (**1**): Yellow amorphous powder (1.1 mg), UV λ_max_ 281, 336 nm; For ^1^H NMR (D_2_O:CD_3_OD, 600 MHz) and ^13^C NMR (D_2_O:CD_3_OD, 150 MHz) spectral assignments, see Table 1. HR-ESI-MS (negative mode): *m/z* 383.0077 [M−H]^−^ (calcd for C_15_H_12_O_10_S, 383.0078 [M−H] ^−^).

Dihydrokaempferol-7-sulfate (**2**): Yellow amorphous powder (1.0 mg), UV λ_max_ 210, 280, 346 nm; For ^1^H NMR (D_2_O:CD_3_OD, 600 MHz) and ^13^C NMR (D_2_O:CD_3_OD, 150 MHz) spectral assignments, see Table 1. HR-ESI-MS (negative mode): *m/z* 367.0130 [M−H]^−^ (calcd for C_15_H_12_O_9_S, 367.0129 [M−H]^−^).

Eriodictyol-7-sulfate (**3**): Yellow amorphous powder (0.87 mg), [α]_25_^D^ +17.8 (c 0.073 in water), UV λ_max_ 281, 336 nm; For ^1^H NMR (D_2_O:CD_3_OD, 600 MHz) and ^13^C NMR (D_2_O:CD_3_OD, 150 MHz) spectral assignments, see Table 1. HR-ESI-MS (negative mode): *m/z* 367.0128 [M−H]^−^ (calcd for C_15_H_12_O_9_S, 367.0129 [M−H]^−^).

Narigenin-7-sulfate (**4**): Colourless amorphous powder (2.9 mg), UV λ_max_ 213, 280, 342 nm; For ^1^H NMR (D_2_O:CD_3_OD, 600 MHz) and ^13^C NMR (D_2_O:CD_3_OD, 150 MHz) spectral assignments, see Table 1. HR-ESI-MS (negative mode): *m/z* 351.0177 [M−H]^−^ (calcd for C_15_H_12_O_8_S, 351.0180 [M−H]^−^).

### 4.5 DPPH radical scavenging assay

The free radical scavenging activity of the samples was evaluated *in vitro* using the 2,2-diphenyl-1-picrylhydrazyl (DPPH) assay following the previously described procedures (Dias et al., 2017). The antioxidant activity of the compounds was expressed as IC_50_, which is the concentration in μM of compound that reduces the concentration of DPPH radicals by 50 %. IC_50_ values were calculated based on three independent experiments.

## References

[bib0005] Barron D., Varin L., Ibrahim R.K., Harborne J.B., Williams C.A. (1988). Sulfated flavonoids—an update. Phytochemistry.

[bib0010] Boeckler G.A., Gershenzon J., Unsicker S.B. (2011). Phenolic glycosides of the Salicaceae and their role as anti-herbivore defences. Phytochemistry.

[bib0015] Correia-da-Silva M., Sousa E., Pinto M.M.M. (2014). Emerging sulfated flavonoids and other polypheols as drugs: nature as an inspiration. Med. Res. Rev..

[bib0020] Dias C.N., Picoli E.A., de T., de Souza G.A., Farag M.A., Scotti M.T., Barbosa-Filho J.M., da Silva M.S., Tavares J.F. (2017). Phenolics metabolism provides a tool for screening drought tolerant *Eucalyptus grandis* hybrids. Aust. J. Crop Sci..

[bib0025] Desborough M.J.R., Keeling D.M. (2017). The aspirin story—from willow to wonder drug. Br. J. Haematol..

[bib0030] Enerstvedt K.H., Lundberg A., Sjøtun I.K., Fadnes P., Jordheim M. (2017). Characterization and seasonal variation of individual flavonoids in *Zostera marina* and *Zostera noltii* from Norwegian coastal waters. Biochem. Syst. Ecol..

[bib0035] Fernandes D.A., Barros R.P.C., Teles Y.C.F., Oliveira L.H.G., Lima J.B., Scotti M.T., Nunes F.C., Conceicao A.S., de Souza M.D.V. (2019). Larvicidal compounds extracted from *Helicteres velutina* K. Schum (Sterculiaceae) evaluated against *Aedes aegypti* L. Molecules.

[bib0040] Gadetskaya A.V., Tarawneh A.H., Zhusupova G.E., Gemejiyeva N.G., Cantrell C.L., Cutler S.J., Ross S.A. (2015). Sulfated phenolic compounds from *Limonium caspium*: isolation, structural elucidation, and biological evaluation. Fitoterapia.

[bib0045] Gidda S.K., Varin L. (2006). Biochemical and molecular characterization of flavonoid 7-sulfotransferase from Arabidopsis thaliana. Plant Physiology and Biochemistry.

[bib0050] Grignon-Dubois M., Rezzonico B. (2018). Phenolic chemistry of the seagrass *Zostera noltei* Hornem. Part 1: first evidence of three infraspecific flavonoid chemotypes in three distinctive geographical regions. Phytochemistry.

[bib0055] Guglielmone H.A., Agnese A.M., Núñez Montoya S.C., Cabrera J.L. (2002). Anticoagulant effect and action mechanism of sulphated flavonoids from *Flaveria bidentis*. Thromb. Res..

[bib0060] Hanley S.J., Karp A. (2014). Genetic strategies for dissecting complex traits in biomass willows (*Salix* spp.). Tree Physiol..

[bib0065] Hashiguchi T., Sakakibara Y., Shimohira T., Kurogi K., Yamasaki M., Nishiyama K., Akashi R., Liu M.-C., Suiko M. (2014). Identification of a novel flavonoid glycoside sulfotransferase in *Arabidopsis thaliana*. J. Biochem..

[bib0070] Ibrahim A.-R.S. (2000). Sulfation of naringenin by *Cunninghamella elegans*. Phytochemistry.

[bib0075] Isebrands J.G., Richardson J. (2014). Poplars and Willows: Trees for Society and the Environment.

[bib0080] Julkunen-Tiitto R., Virjamo V., Arimura G., Maffei M. (2016). Biosynthesis and roles of salicaceae salicylates. Plant Specialized Metabolism. Genomics, Biochemistry, and Biological Functions.

[bib0085] Lauron-Moreau A., Pitre F.E., Argus G.W., Labrecque M., Brouillet L. (2015). Phylogenetic relationships of American willows (*Salix* l., Salicaceae). PLoS One.

[bib0090] Li H.-W., Zou T.-B., Jia Q., Xia E.-Q., Cao W.-J., Liu W., He T.-P., Wang Q. (2016). Anticancer effects of morin-7-sulphate sodium, a flavonoid derivative, in mouse melanoma cells. Biomed. Pharmacother..

[bib0095] Meng S., Wu B., Singh R., Yin T., Morrow J.K., Zhang S., Hu M. (2012). SULT1A3-mediated regiospecific 7-*O*-sulfation of flavonoids in Caco-2 cells can be explained by the relevant molecular docking studies. Mol. Pharm..

[bib0100] Nguyen P.-D., Sayagh C., Borie N., Lavaud C. (2017). Anti-radical flavonol glycosides from the aerial parts of *Cleome chelidonii* L.f. Phytochemistry.

[bib0105] Noleto-Dias C., Ward J.L., Bellisai A., Lomax C., Beale M.H. (2018). Salicin-7-sulfate: a new salicinoid from willow and implications for herbal medicine. Fitoterapia.

[bib0110] Nyman T., Julkunen-Tiitto R. (2005). Chemical variation within and among six northern willow species. Phytochemistry.

[bib0115] Op de Beck P., Dijoux M.-G., Cartier G., Mariotte A.-M. (1998). Quercitrin 3′-sulphate from leaves of *Leea guinensis*. Phytochemistry.

[bib0120] Pietta G.-P. (2000). Flavonoids as antioxidants. J. Nat. Prod..

[bib0125] Ragan M.A. (1978). Phenol sulfate esters: ultraviolet, infrared, ^1^H and ^13^C nuclear magnetic resonance spectroscopic investigation. Can. J. Chem..

[bib0130] Roubalová L., Purchartová K., Papoušková B., Vacek J., Křen V., Ulrichová J., Vrba J. (2015). Sulfation modulates the cell uptake, antiradical activity and biological effects of flavonoids in vitro: an examination of quercetin, isoquercitrin and taxifolin. Bioorg. Med. Chem..

[bib0135] Schmidt J. (2016). Negative ion electrospray high-resolution tandem mass spectrometry of polyphenols. J. Mass Spectrom..

[bib0140] Teles Y.C.F., Horta C.C.R., Agra M.D., Siheri W., Boyd M., Igoli J.O., Gray A.I., de Souza M.D.V. (2015). New sulphated flavonoids from *Wissadula periplocifolia* (L.) C. Presl (Malvaceae). Molecules.

[bib0145] Teles Y.C.F., Souza M.S.R., de Souza M.F.V. (2018). Sulphated flavonoids: biosynthesis, structures and biological activities. Molecules.

[bib0150] Vacek J., Papoušková B., Vrba J., Zatloukalová M., Křen V., Ulrichová J. (2013). LC–MS metabolic study on quercetin and taxifolin galloyl esters using human hepatocytes as toxicity and biotransformation *in vitro* cell model. J. Pharm. Biomed. Anal..

[bib0155] Wiesneth S., Aas G., Heilmann J., Jürgenliemk G. (2018). Investigation of the flavan-3-ol patterns in willow species during one growing-season. Phytochemistry.

[bib0160] Yang P., Xu F., Li H.-F., Wang Y., Li F.-C., Shang M.-Y., Liu G.-X., Wang X., Cai S.-Q. (2016). Detection of 191 taxifolin metabolites and their distribution in rats using HPLC-ESI-IT-TOF-MSn. Molecules.

