# Supplementary material for: Sulfated flavanones and dihydroflavonols from willow
Source: Phytochem Lett. 2020 Feb;35:88–93. doi: 10.1016/j.phytol.2019.11.008 (PMC6988443; doi:10.1016/j.phytol.2019.11.008)
Supplement: Supplementary file 1 [file mmc1.pptx]

## Slide 1
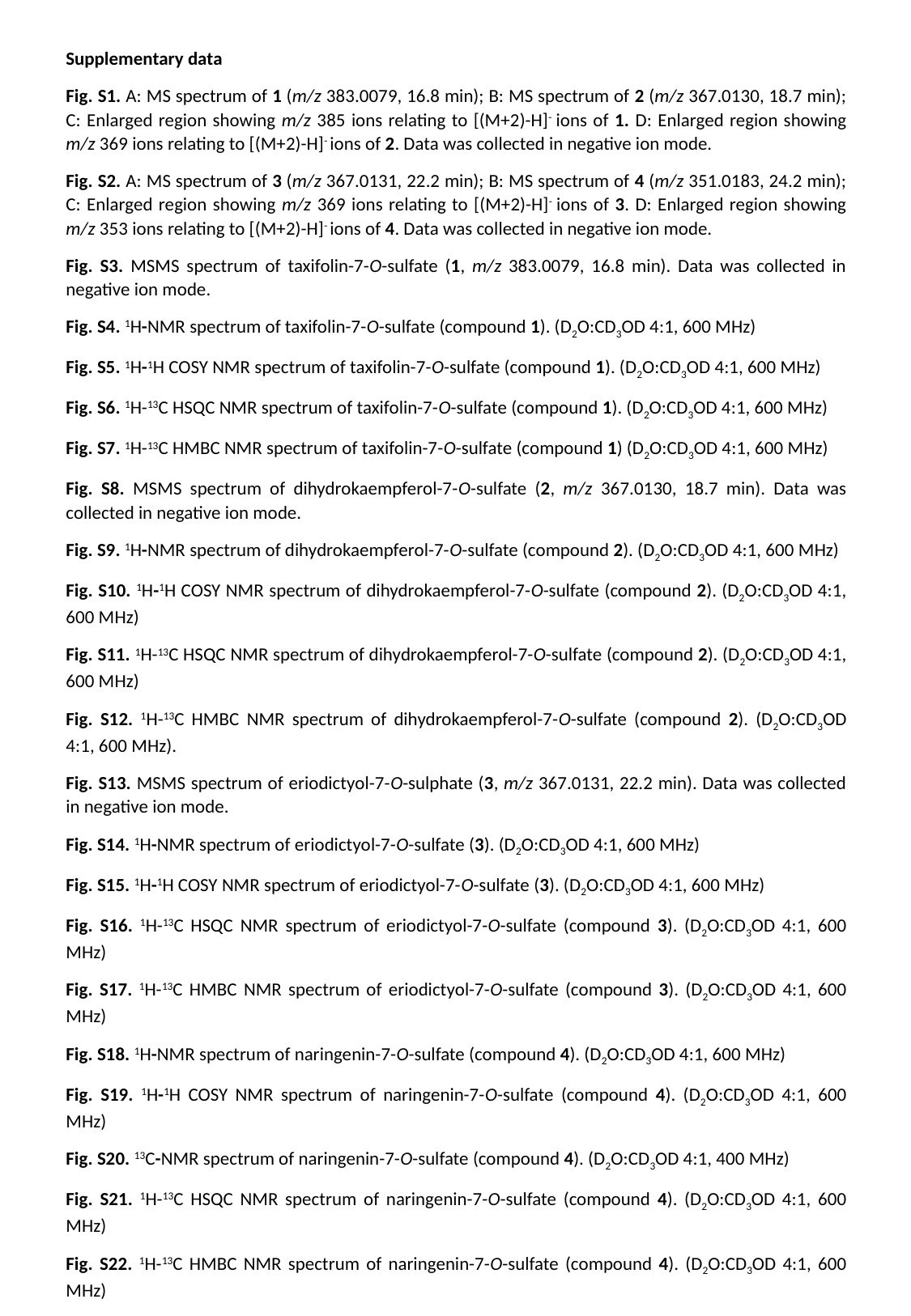

Supplementary data
Fig. S1. A: MS spectrum of 1 (m/z 383.0079, 16.8 min); B: MS spectrum of 2 (m/z 367.0130, 18.7 min); C: Enlarged region showing m/z 385 ions relating to [(M+2)-H]- ions of 1. D: Enlarged region showing m/z 369 ions relating to [(M+2)-H]- ions of 2. Data was collected in negative ion mode.
Fig. S2. A: MS spectrum of 3 (m/z 367.0131, 22.2 min); B: MS spectrum of 4 (m/z 351.0183, 24.2 min); C: Enlarged region showing m/z 369 ions relating to [(M+2)-H]- ions of 3. D: Enlarged region showing m/z 353 ions relating to [(M+2)-H]- ions of 4. Data was collected in negative ion mode.
Fig. S3. MSMS spectrum of taxifolin-7-O-sulfate (1, m/z 383.0079, 16.8 min). Data was collected in negative ion mode.
Fig. S4. 1H-NMR spectrum of taxifolin-7-O-sulfate (compound 1). (D2O:CD3OD 4:1, 600 MHz)
Fig. S5. 1H-1H COSY NMR spectrum of taxifolin-7-O-sulfate (compound 1). (D2O:CD3OD 4:1, 600 MHz)
Fig. S6. 1H-13C HSQC NMR spectrum of taxifolin-7-O-sulfate (compound 1). (D2O:CD3OD 4:1, 600 MHz)
Fig. S7. 1H-13C HMBC NMR spectrum of taxifolin-7-O-sulfate (compound 1) (D2O:CD3OD 4:1, 600 MHz)
Fig. S8. MSMS spectrum of dihydrokaempferol-7-O-sulfate (2, m/z 367.0130, 18.7 min). Data was collected in negative ion mode.
Fig. S9. 1H-NMR spectrum of dihydrokaempferol-7-O-sulfate (compound 2). (D2O:CD3OD 4:1, 600 MHz)
Fig. S10. 1H-1H COSY NMR spectrum of dihydrokaempferol-7-O-sulfate (compound 2). (D2O:CD3OD 4:1, 600 MHz)
Fig. S11. 1H-13C HSQC NMR spectrum of dihydrokaempferol-7-O-sulfate (compound 2). (D2O:CD3OD 4:1, 600 MHz)
Fig. S12. 1H-13C HMBC NMR spectrum of dihydrokaempferol-7-O-sulfate (compound 2). (D2O:CD3OD 4:1, 600 MHz).
Fig. S13. MSMS spectrum of eriodictyol-7-O-sulphate (3, m/z 367.0131, 22.2 min). Data was collected in negative ion mode.
Fig. S14. 1H-NMR spectrum of eriodictyol-7-O-sulfate (3). (D2O:CD3OD 4:1, 600 MHz)
Fig. S15. 1H-1H COSY NMR spectrum of eriodictyol-7-O-sulfate (3). (D2O:CD3OD 4:1, 600 MHz)
Fig. S16. 1H-13C HSQC NMR spectrum of eriodictyol-7-O-sulfate (compound 3). (D2O:CD3OD 4:1, 600 MHz)
Fig. S17. 1H-13C HMBC NMR spectrum of eriodictyol-7-O-sulfate (compound 3). (D2O:CD3OD 4:1, 600 MHz)
Fig. S18. 1H-NMR spectrum of naringenin-7-O-sulfate (compound 4). (D2O:CD3OD 4:1, 600 MHz)
Fig. S19. 1H-1H COSY NMR spectrum of naringenin-7-O-sulfate (compound 4). (D2O:CD3OD 4:1, 600 MHz)
Fig. S20. 13C-NMR spectrum of naringenin-7-O-sulfate (compound 4). (D2O:CD3OD 4:1, 400 MHz)
Fig. S21. 1H-13C HSQC NMR spectrum of naringenin-7-O-sulfate (compound 4). (D2O:CD3OD 4:1, 600 MHz)
Fig. S22. 1H-13C HMBC NMR spectrum of naringenin-7-O-sulfate (compound 4). (D2O:CD3OD 4:1, 600 MHz)

## Slide 2
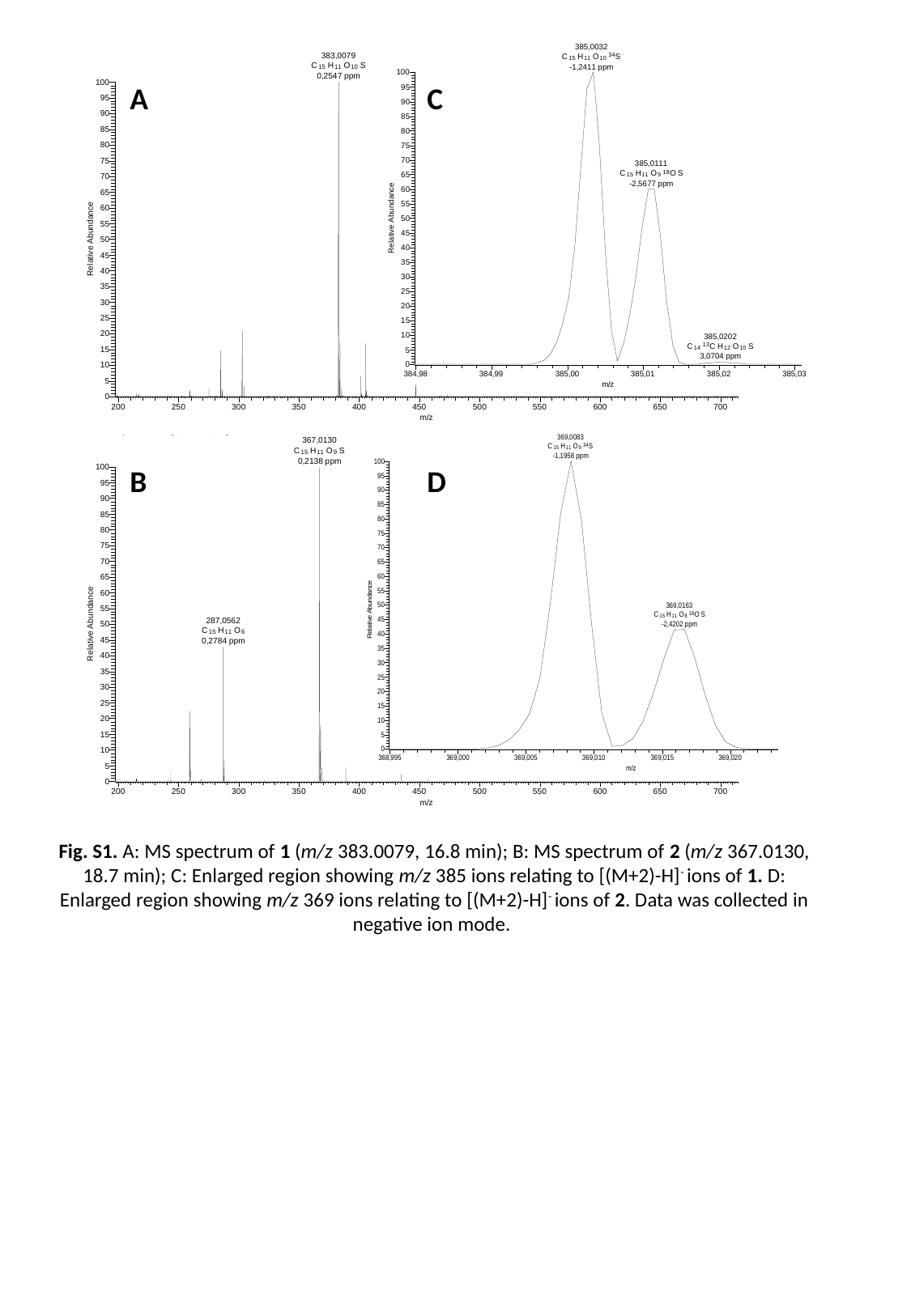

A
C
B
D
Fig. S1. A: MS spectrum of 1 (m/z 383.0079, 16.8 min); B: MS spectrum of 2 (m/z 367.0130, 18.7 min); C: Enlarged region showing m/z 385 ions relating to [(M+2)-H]- ions of 1. D: Enlarged region showing m/z 369 ions relating to [(M+2)-H]- ions of 2. Data was collected in negative ion mode.

## Slide 3
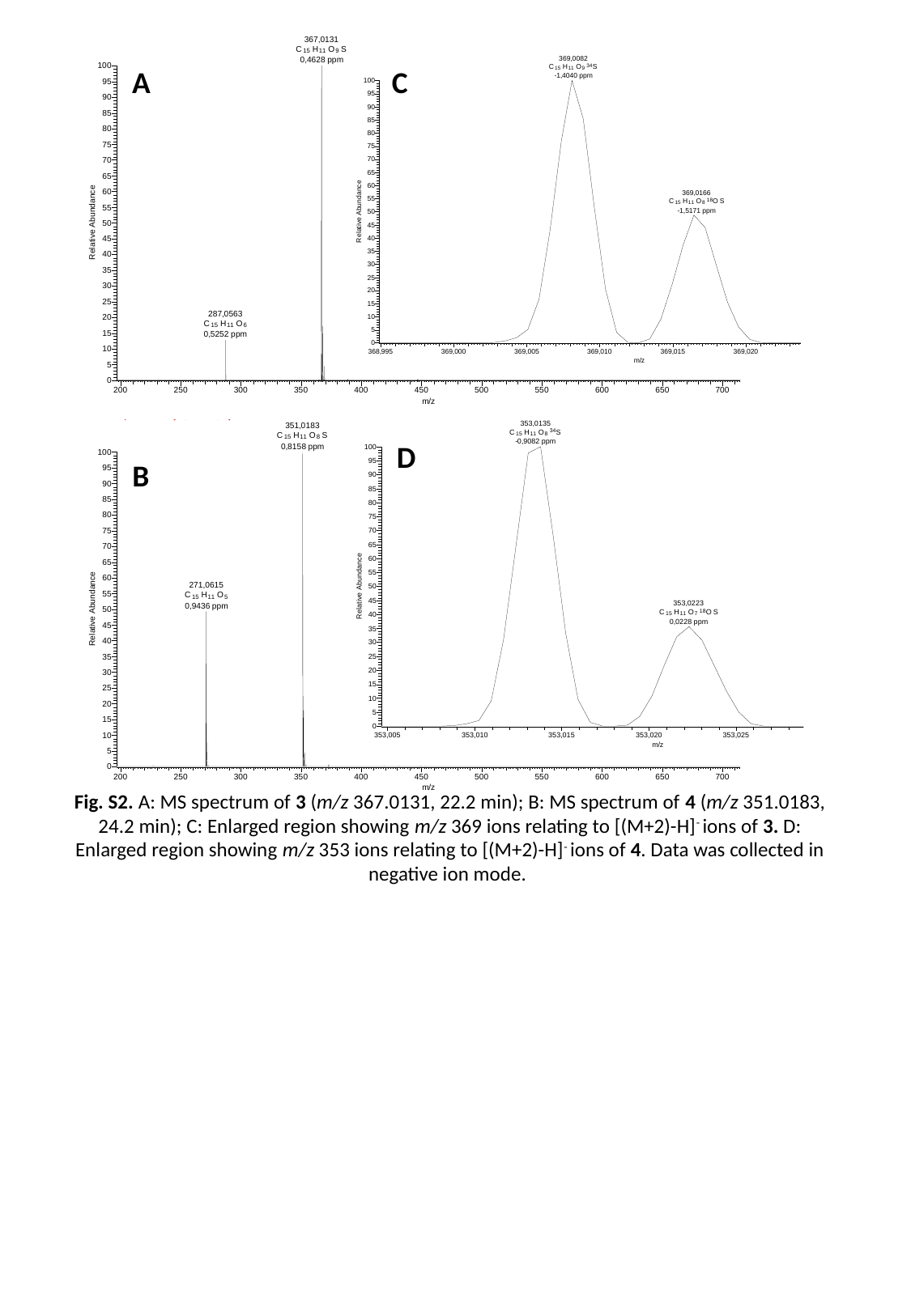

A
C
D
B
Fig. S2. A: MS spectrum of 3 (m/z 367.0131, 22.2 min); B: MS spectrum of 4 (m/z 351.0183, 24.2 min); C: Enlarged region showing m/z 369 ions relating to [(M+2)-H]- ions of 3. D: Enlarged region showing m/z 353 ions relating to [(M+2)-H]- ions of 4. Data was collected in negative ion mode.

## Slide 4
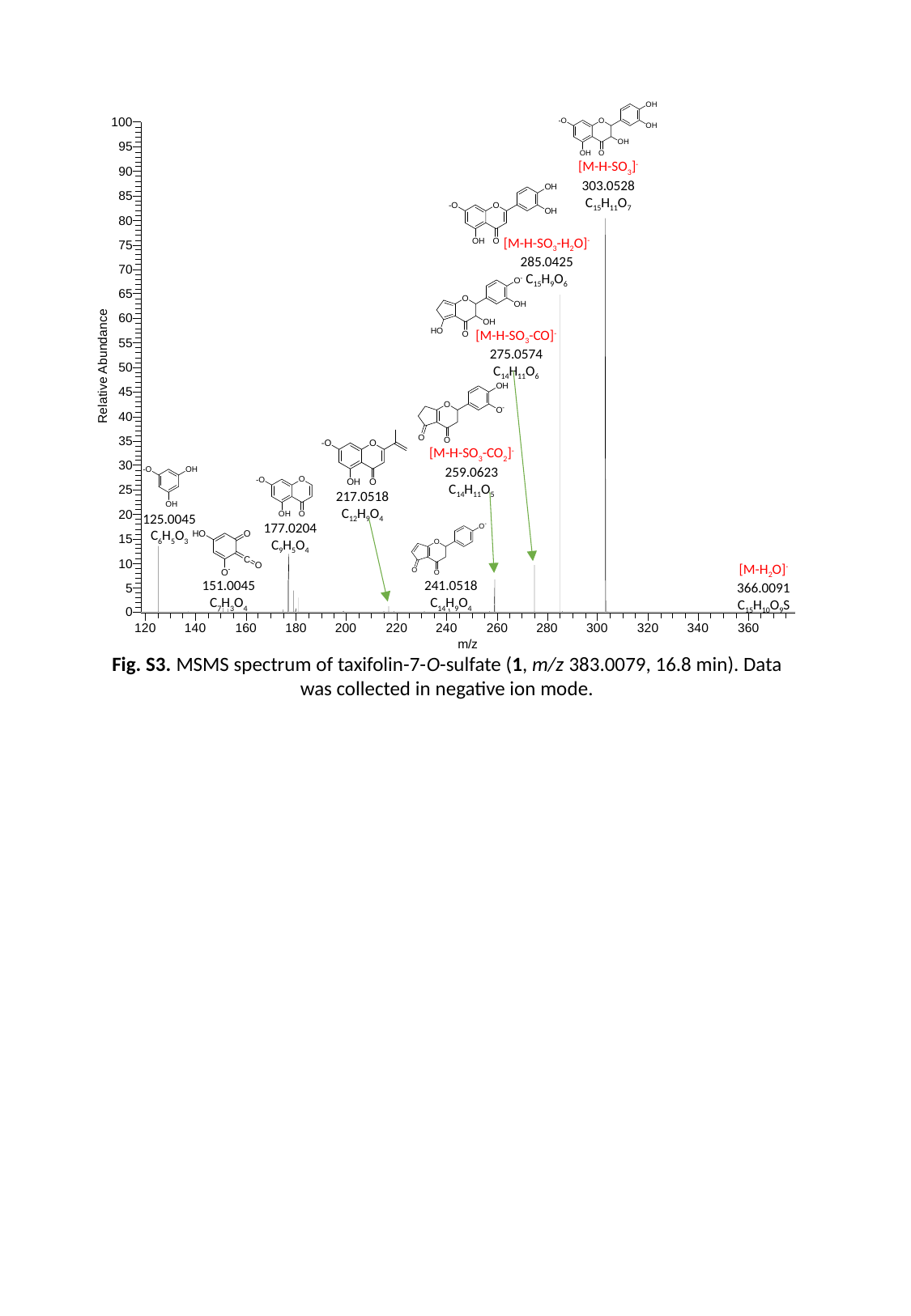

[M-H-SO3]-
303.0528
C15H11O7
[M-H-SO3-H2O]-
285.0425
C15H9O6
[M-H-SO3-CO]-
275.0574
C14H11O6
[M-H-SO3-CO2]-
259.0623
C14H11O5
217.0518
C12H9O4
125.0045
C6H5O3
177.0204
C9H5O4
[M-H2O]-
366.0091
C15H10O9S
151.0045
C7H3O4
241.0518
C14H9O4
Fig. S3. MSMS spectrum of taxifolin-7-O-sulfate (1, m/z 383.0079, 16.8 min). Data was collected in negative ion mode.

## Slide 5
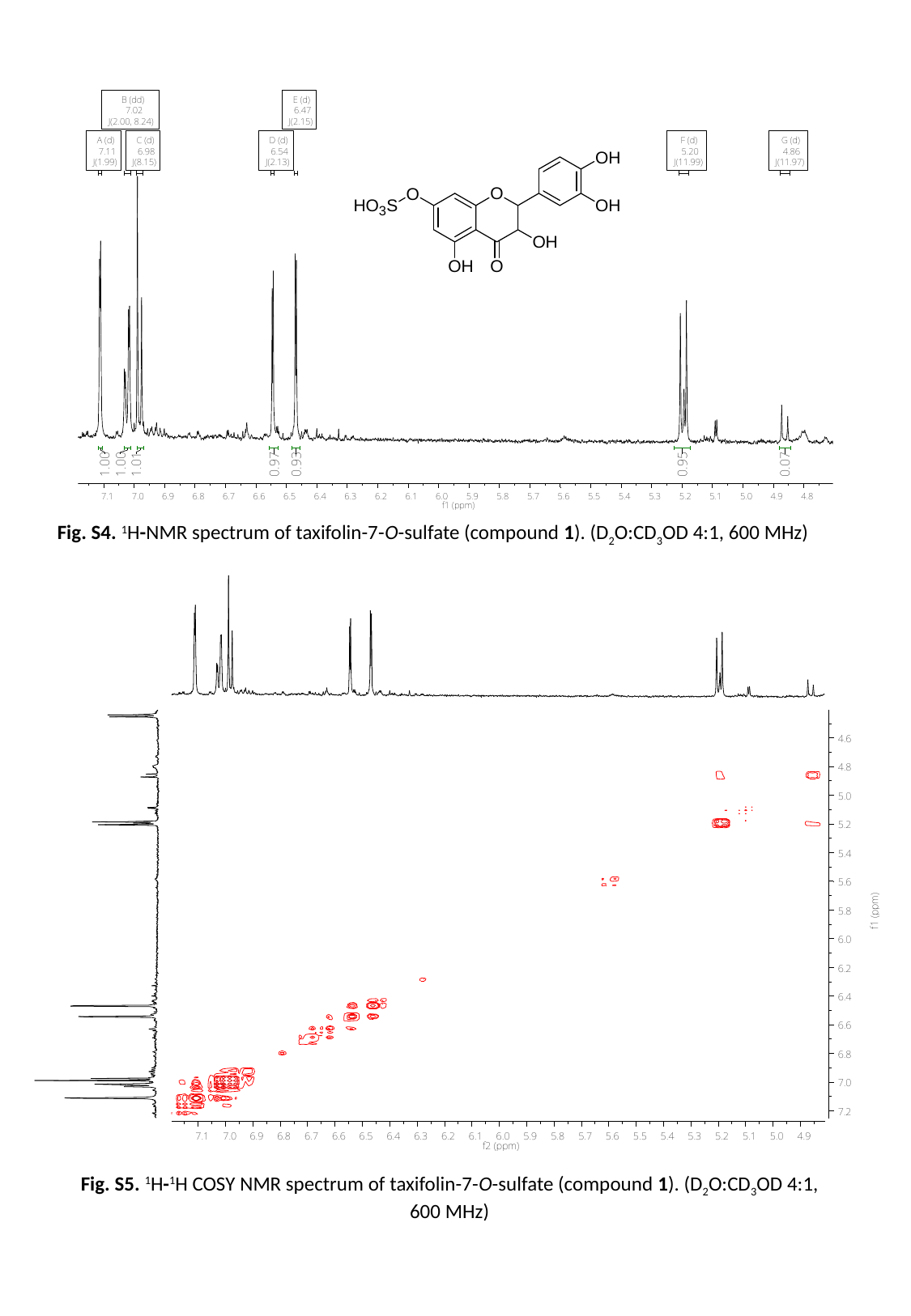

Fig. S4. 1H-NMR spectrum of taxifolin-7-O-sulfate (compound 1). (D2O:CD3OD 4:1, 600 MHz)
Fig. S5. 1H-1H COSY NMR spectrum of taxifolin-7-O-sulfate (compound 1). (D2O:CD3OD 4:1, 600 MHz)

## Slide 6
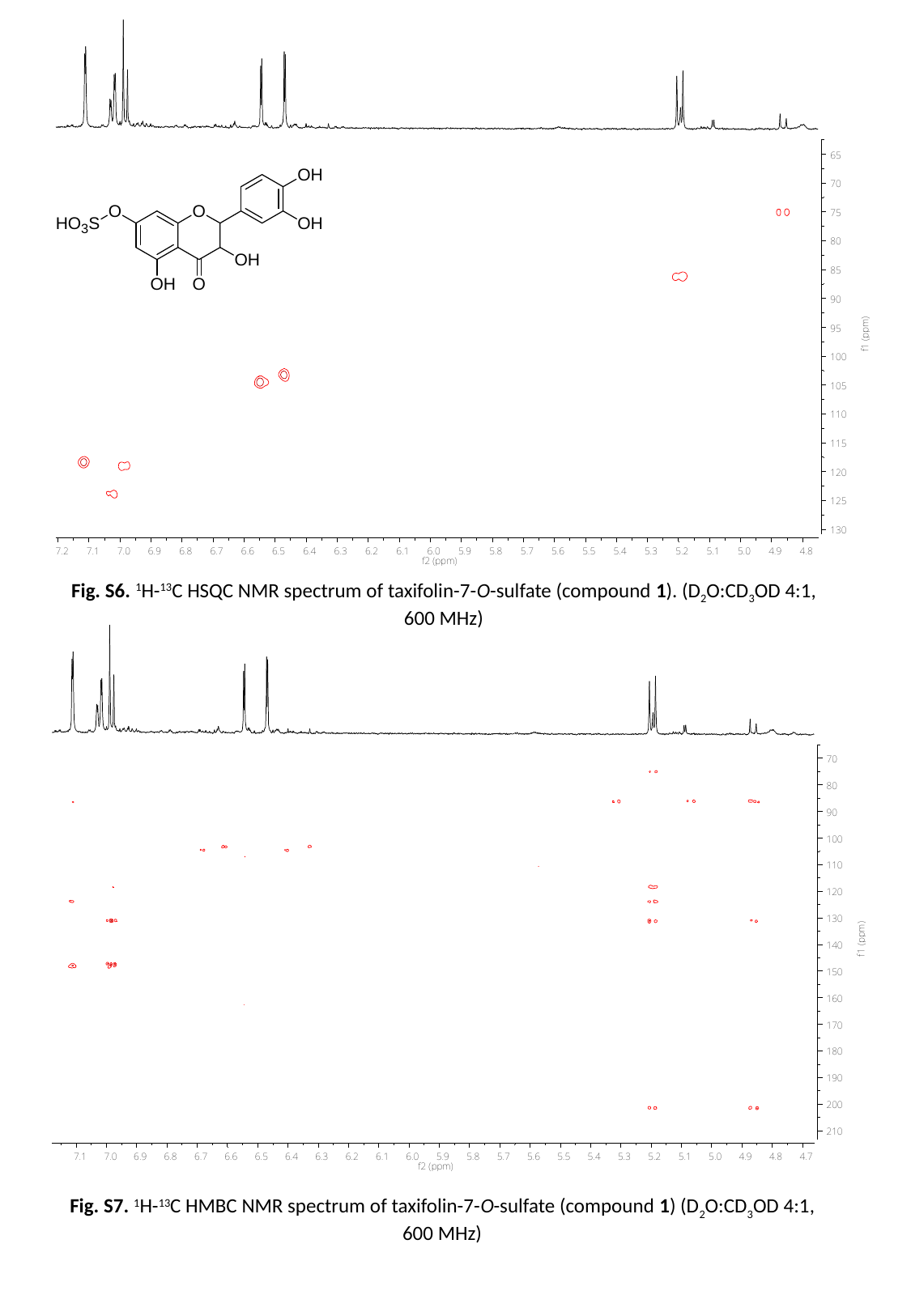

Fig. S6. 1H-13C HSQC NMR spectrum of taxifolin-7-O-sulfate (compound 1). (D2O:CD3OD 4:1, 600 MHz)
Fig. S7. 1H-13C HMBC NMR spectrum of taxifolin-7-O-sulfate (compound 1) (D2O:CD3OD 4:1, 600 MHz)

## Slide 7
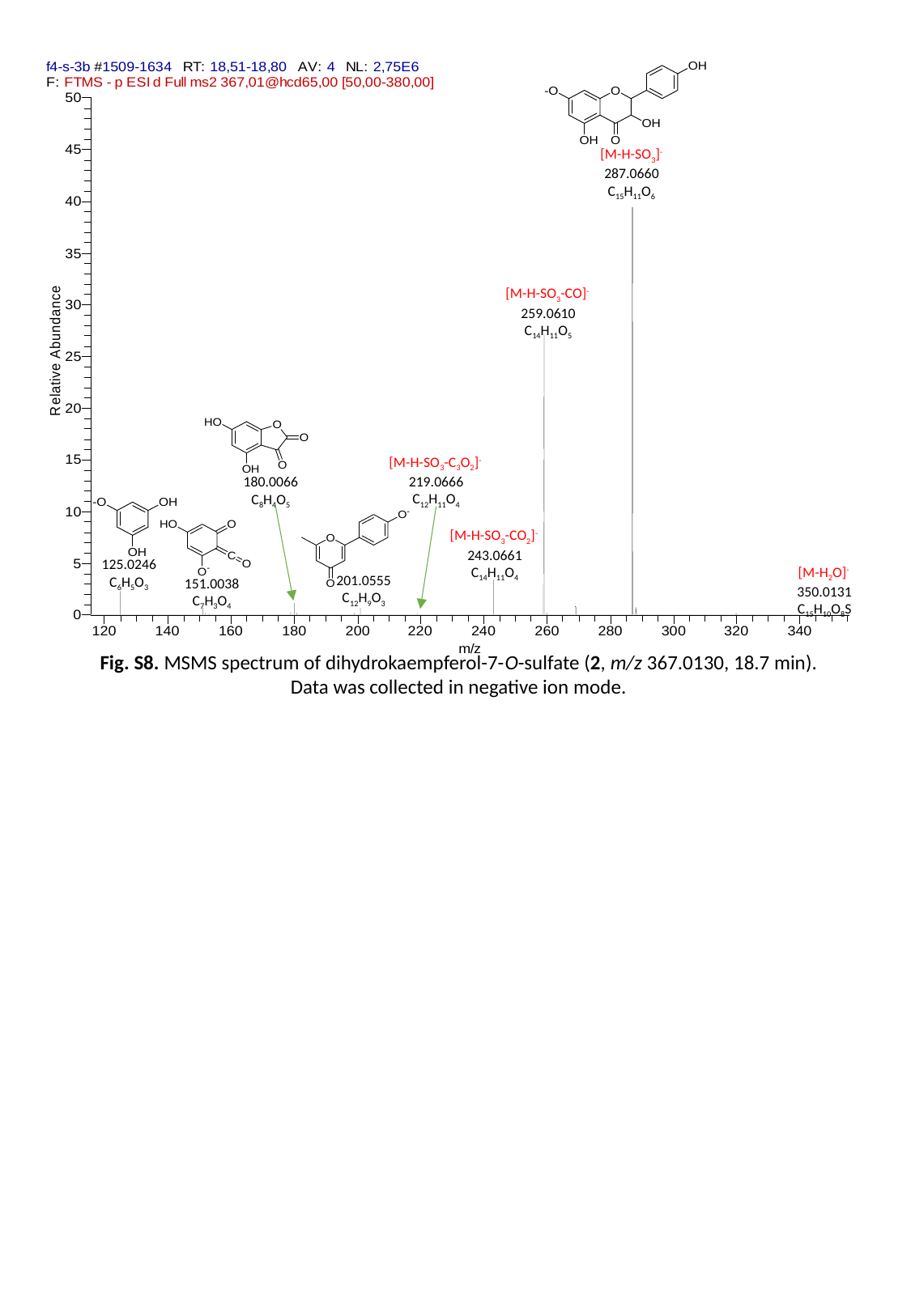

[M-H-SO3]-
287.0660
C15H11O6
[M-H-SO3-CO]-
259.0610
C14H11O5
[M-H-SO3-C3O2]-
219.0666
C12H11O4
180.0066
C8H4O5
[M-H-SO3-CO2]-
243.0661
C14H11O4
125.0246
C6H5O3
[M-H2O]-
350.0131
C15H10O8S
201.0555
C12H9O3
151.0038
C7H3O4
Fig. S8. MSMS spectrum of dihydrokaempferol-7-O-sulfate (2, m/z 367.0130, 18.7 min). Data was collected in negative ion mode.

## Slide 8
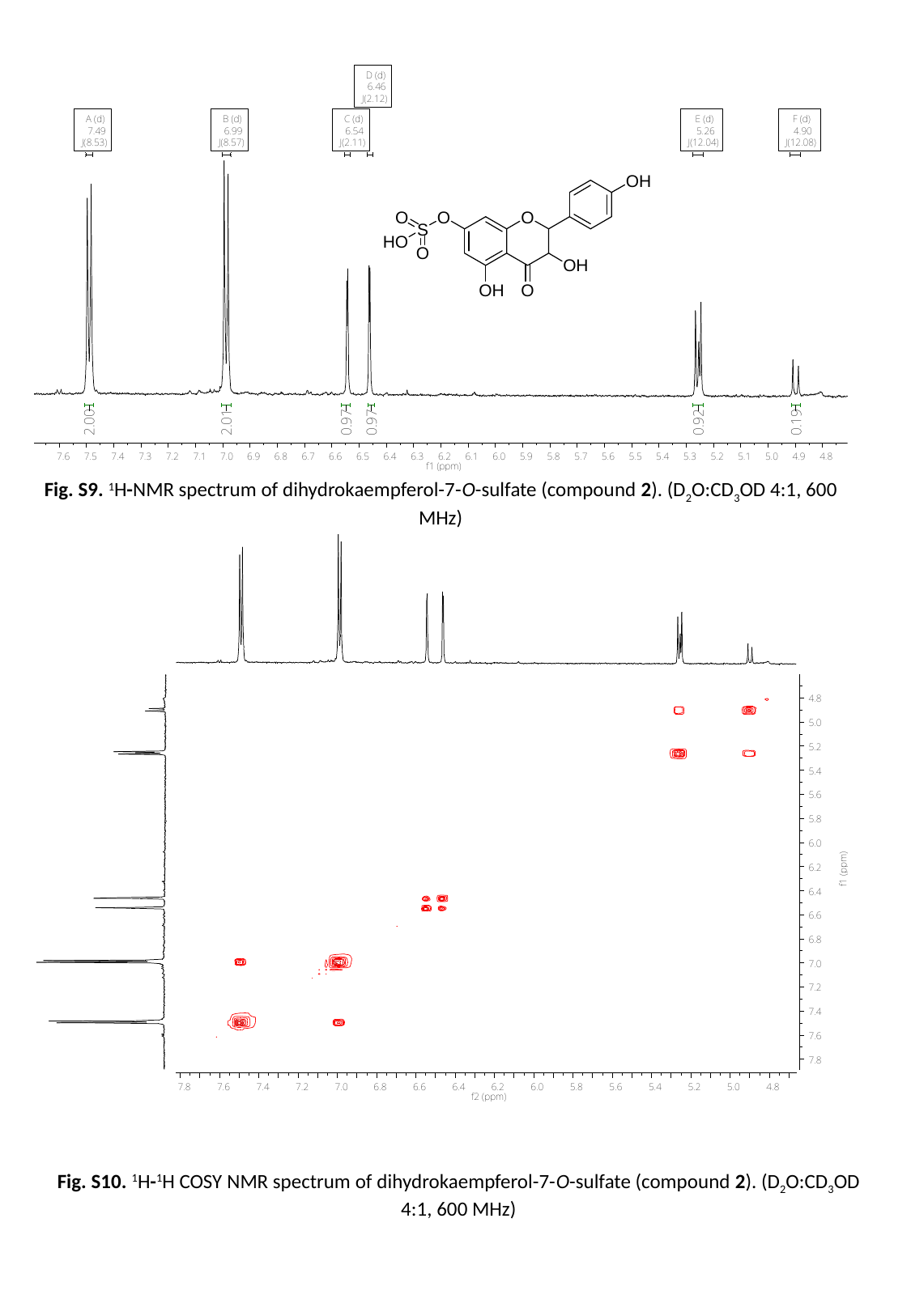

Fig. S9. 1H-NMR spectrum of dihydrokaempferol-7-O-sulfate (compound 2). (D2O:CD3OD 4:1, 600 MHz)
Fig. S10. 1H-1H COSY NMR spectrum of dihydrokaempferol-7-O-sulfate (compound 2). (D2O:CD3OD 4:1, 600 MHz)

## Slide 9
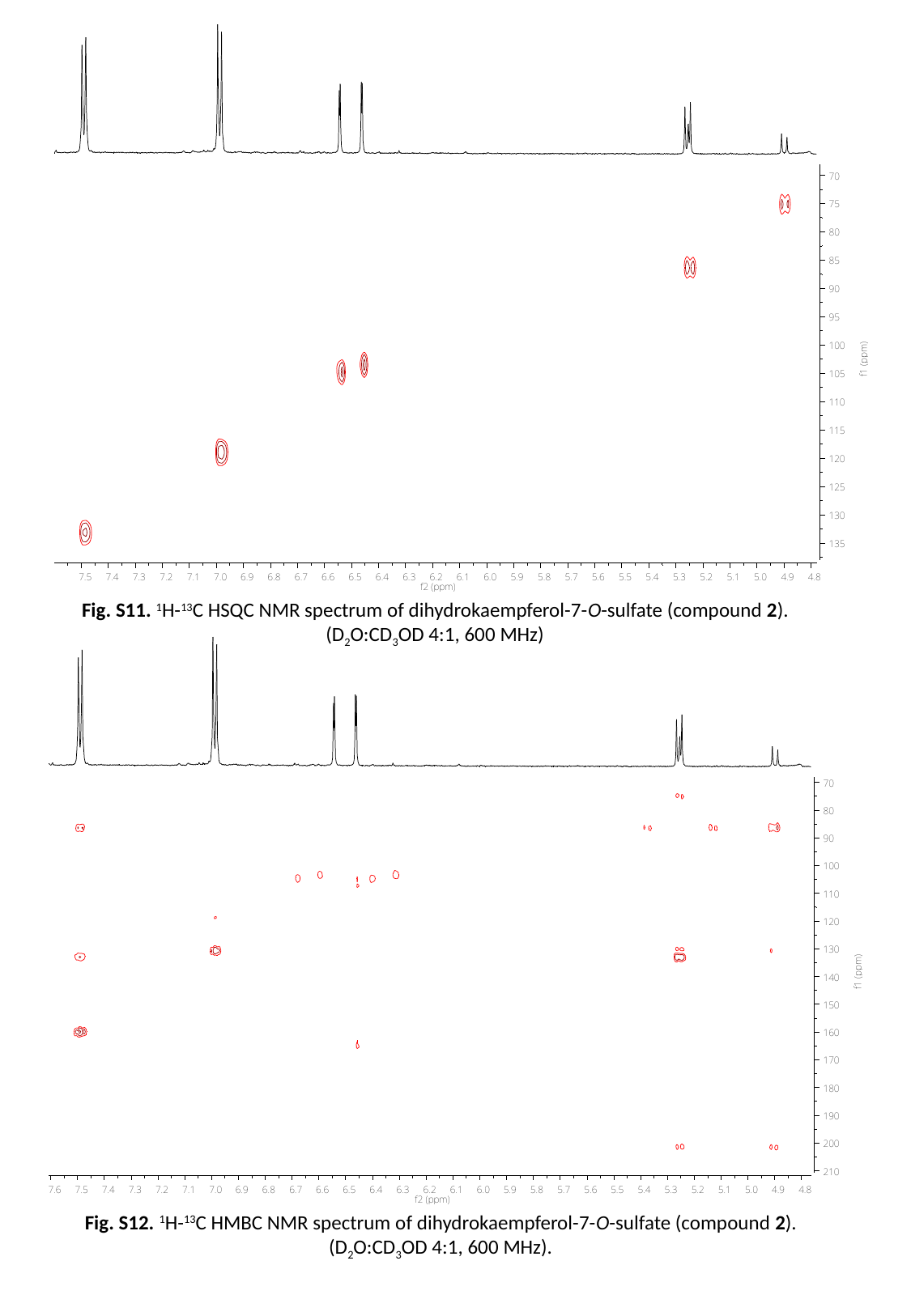

Fig. S11. 1H-13C HSQC NMR spectrum of dihydrokaempferol-7-O-sulfate (compound 2). (D2O:CD3OD 4:1, 600 MHz)
Fig. S12. 1H-13C HMBC NMR spectrum of dihydrokaempferol-7-O-sulfate (compound 2). (D2O:CD3OD 4:1, 600 MHz).

## Slide 10
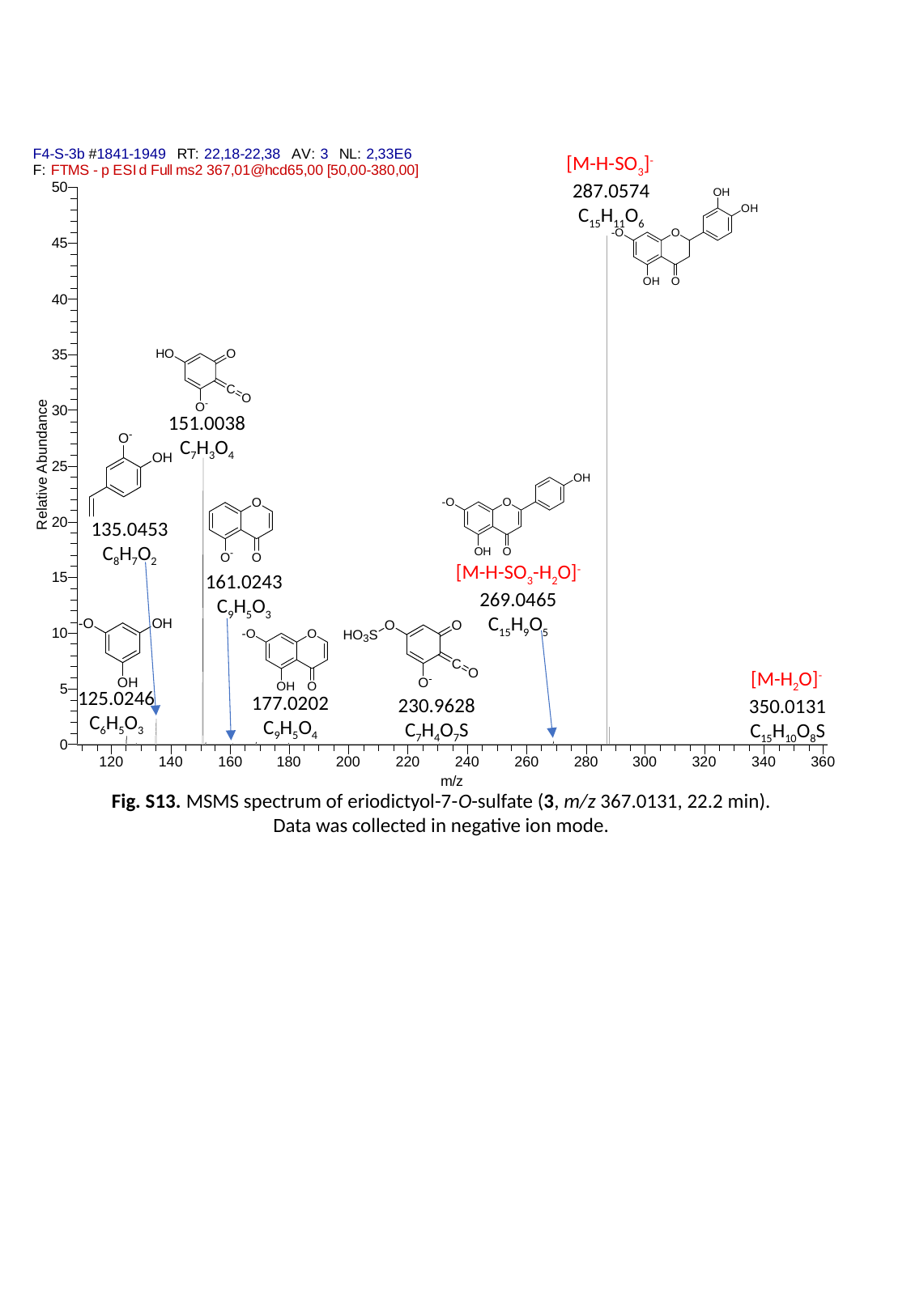

[M-H-SO3]-
287.0574
C15H11O6
151.0038
C7H3O4
135.0453
C8H7O2
[M-H-SO3-H2O]-
269.0465
C15H9O5
161.0243
C9H5O3
[M-H2O]-
350.0131
C15H10O8S
125.0246
C6H5O3
177.0202
C9H5O4
230.9628
C7H4O7S
Fig. S13. MSMS spectrum of eriodictyol-7-O-sulfate (3, m/z 367.0131, 22.2 min). Data was collected in negative ion mode.

## Slide 11
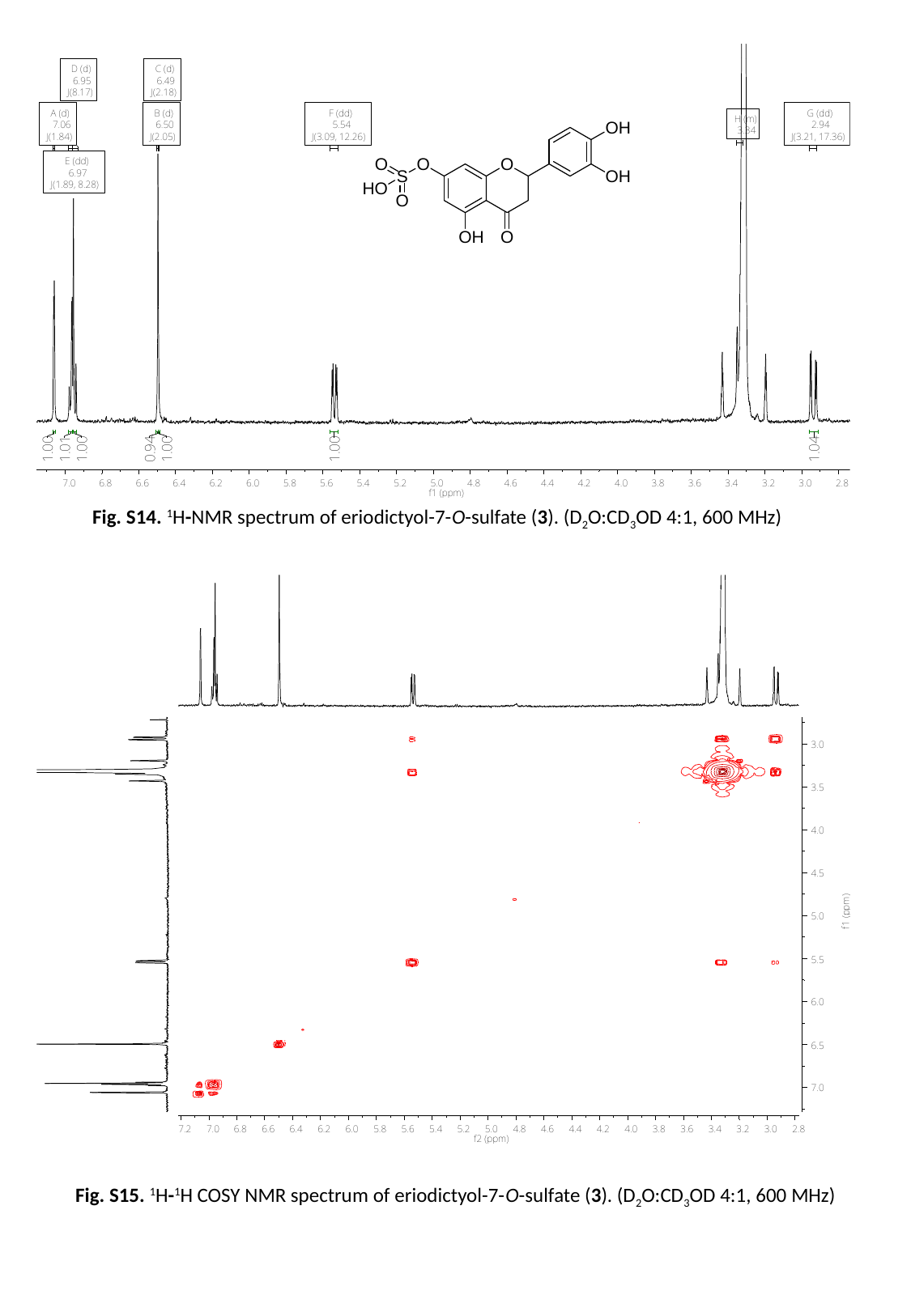

Fig. S14. 1H-NMR spectrum of eriodictyol-7-O-sulfate (3). (D2O:CD3OD 4:1, 600 MHz)
Fig. S15. 1H-1H COSY NMR spectrum of eriodictyol-7-O-sulfate (3). (D2O:CD3OD 4:1, 600 MHz)

## Slide 12
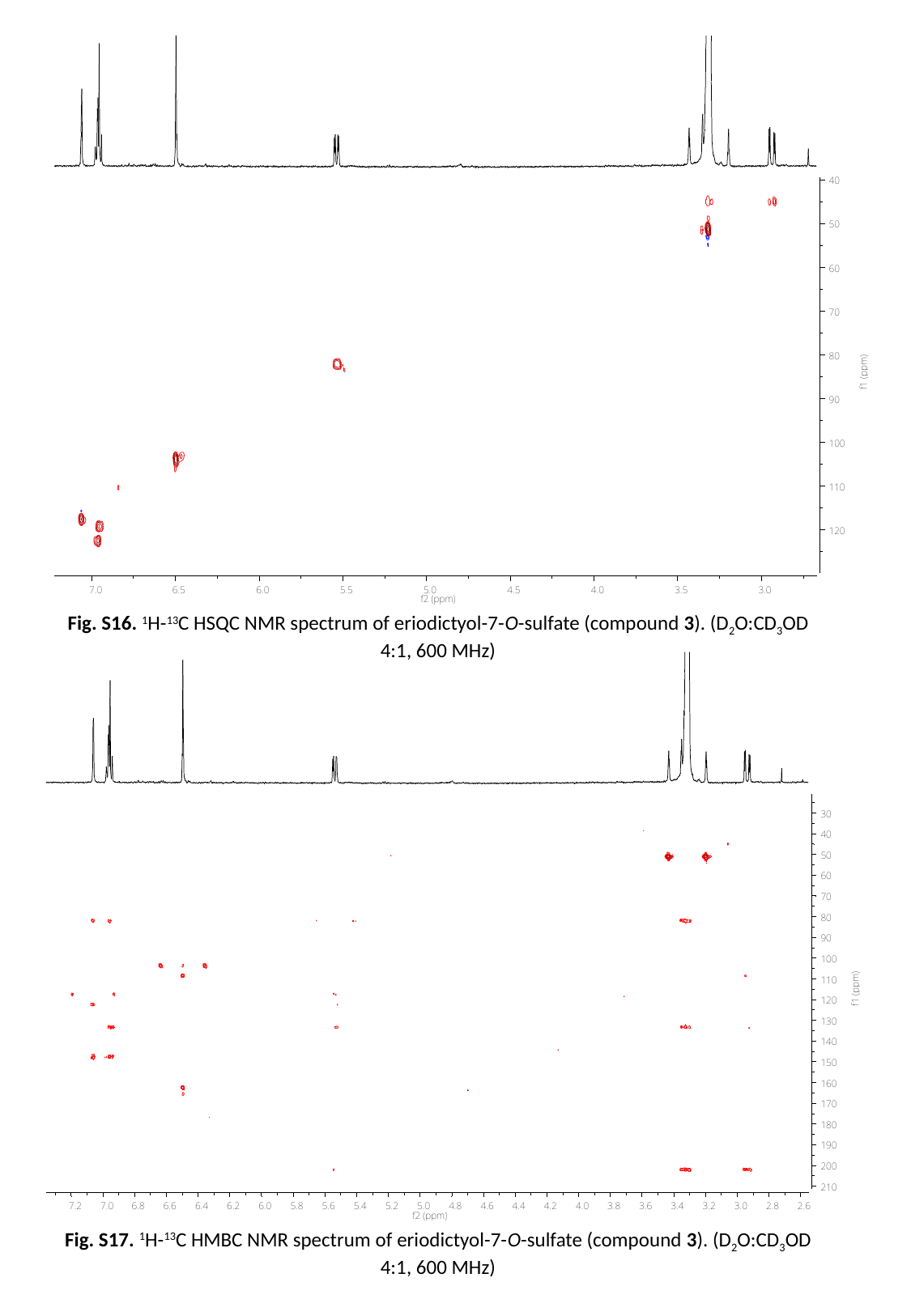

Fig. S16. 1H-13C HSQC NMR spectrum of eriodictyol-7-O-sulfate (compound 3). (D2O:CD3OD 4:1, 600 MHz)
Fig. S17. 1H-13C HMBC NMR spectrum of eriodictyol-7-O-sulfate (compound 3). (D2O:CD3OD 4:1, 600 MHz)

## Slide 13
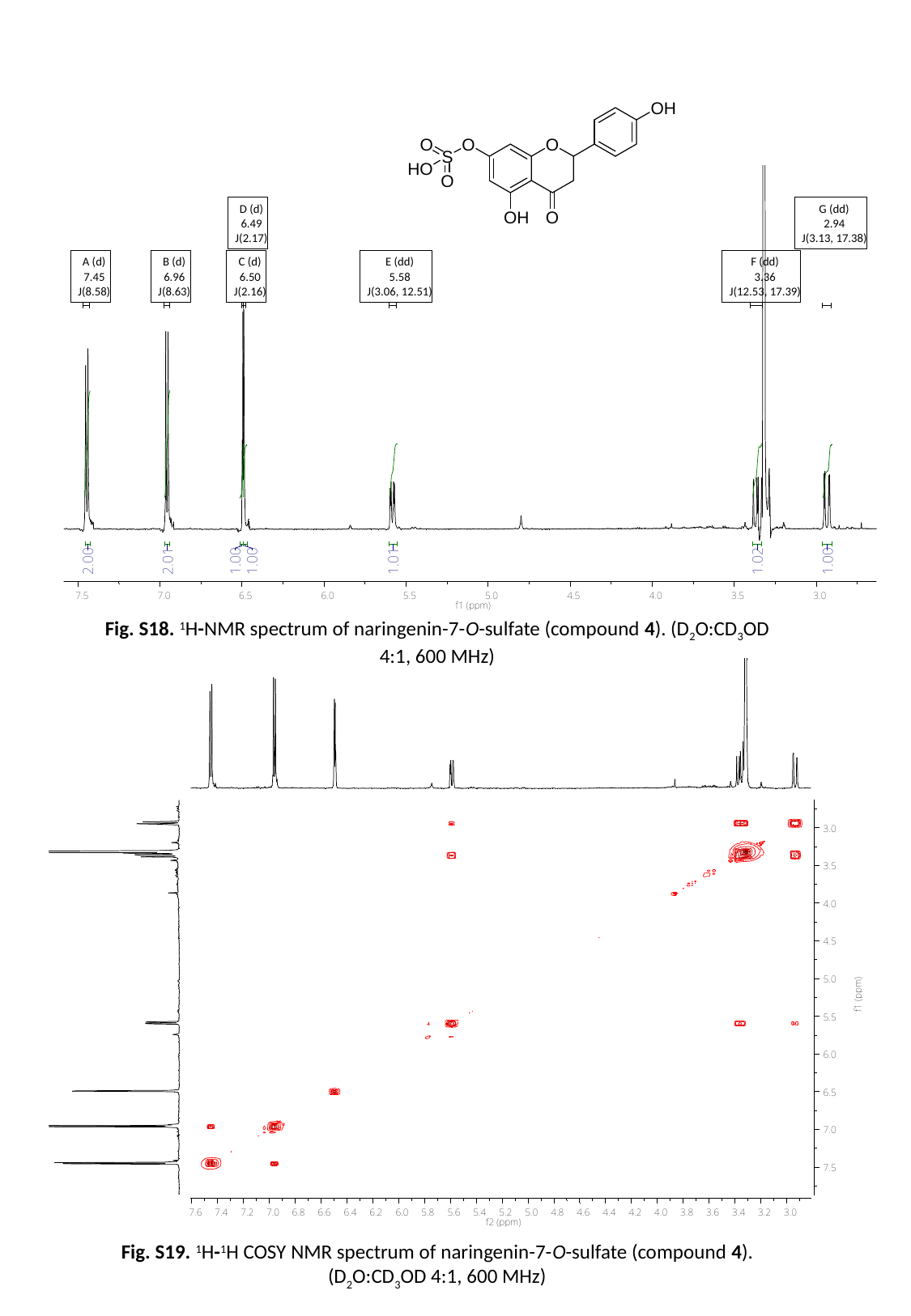

Fig. S18. 1H-NMR spectrum of naringenin-7-O-sulfate (compound 4). (D2O:CD3OD 4:1, 600 MHz)
Fig. S19. 1H-1H COSY NMR spectrum of naringenin-7-O-sulfate (compound 4). (D2O:CD3OD 4:1, 600 MHz)

## Slide 14
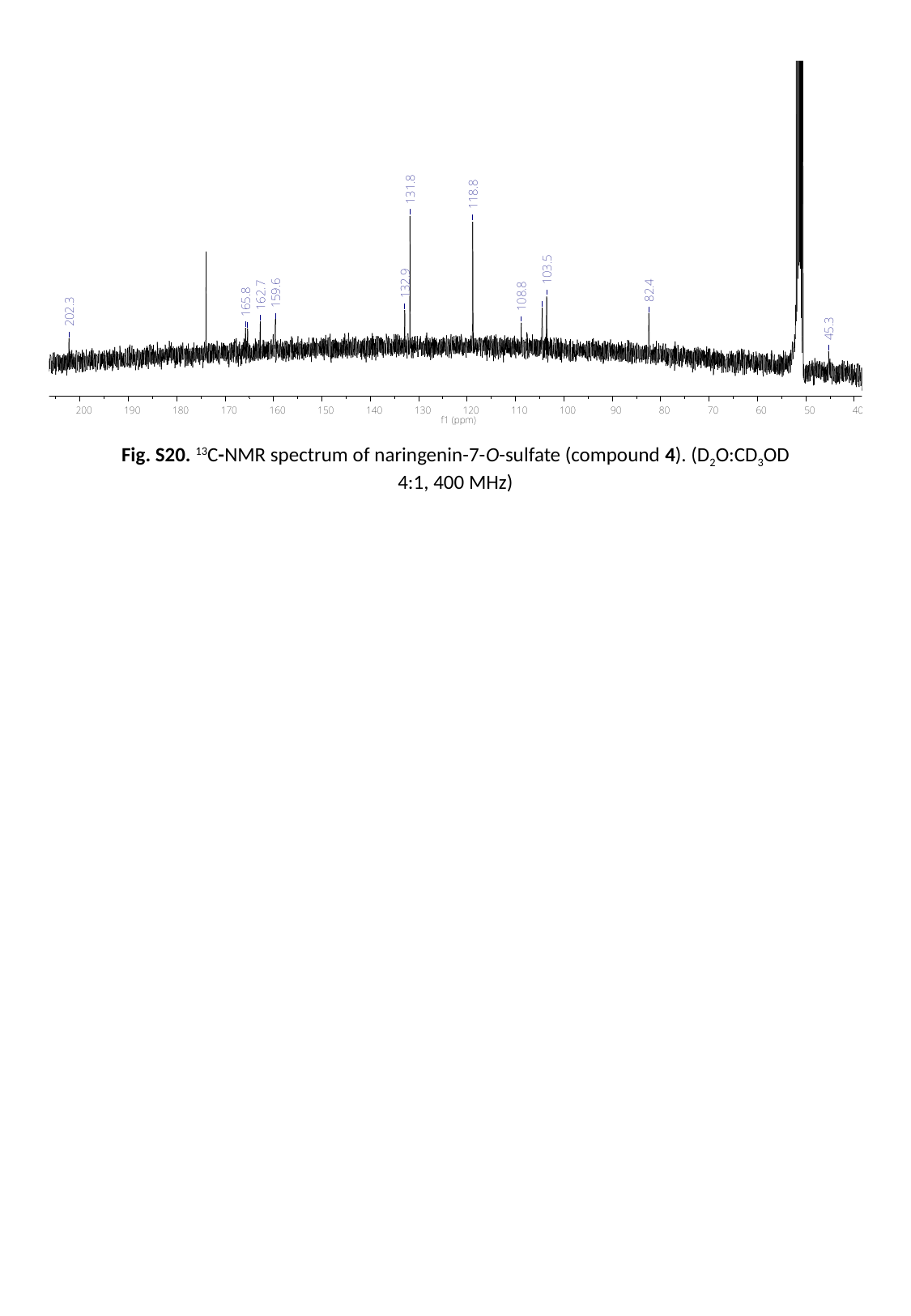

Fig. S20. 13C-NMR spectrum of naringenin-7-O-sulfate (compound 4). (D2O:CD3OD 4:1, 400 MHz)

## Slide 15
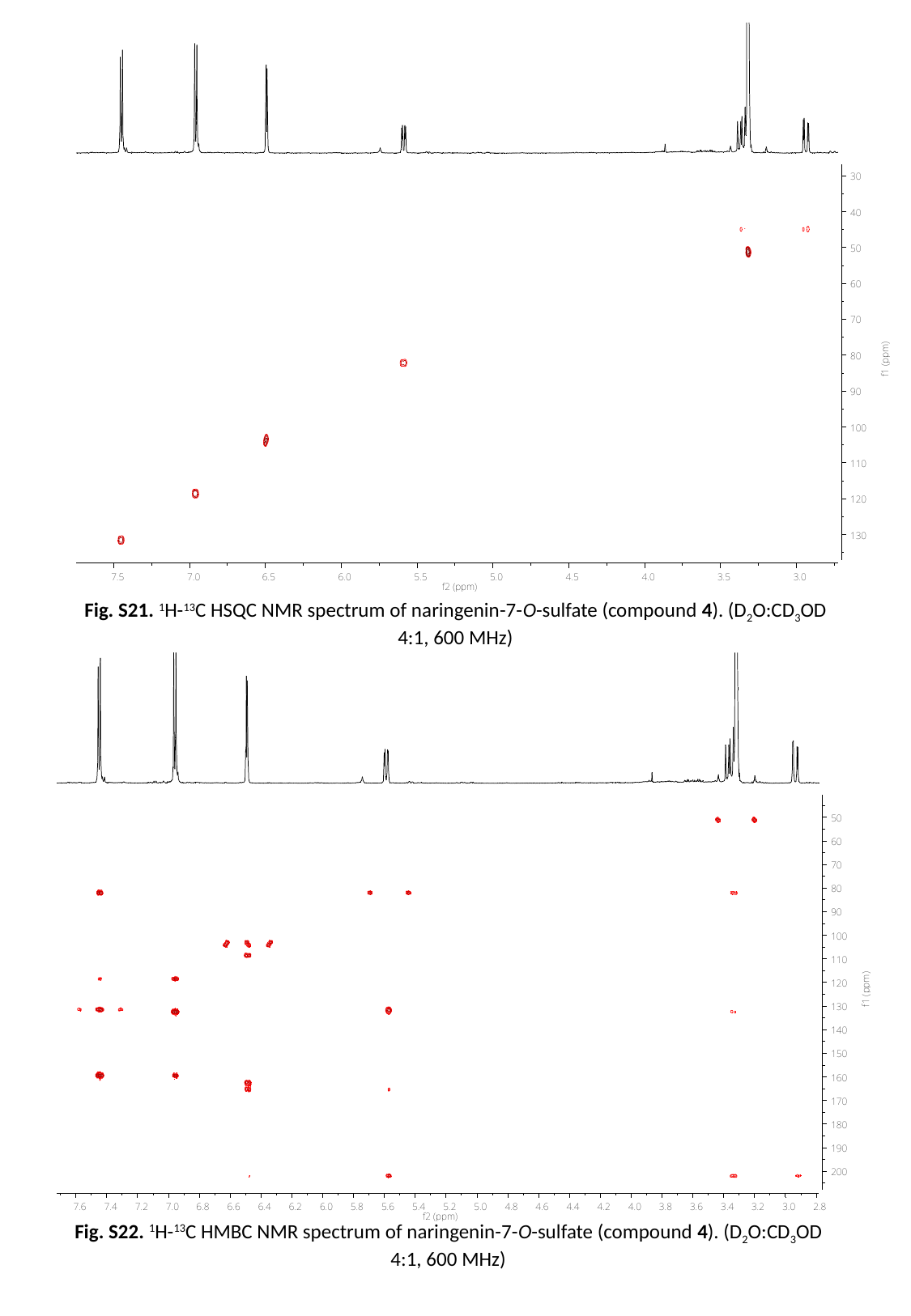

Fig. S21. 1H-13C HSQC NMR spectrum of naringenin-7-O-sulfate (compound 4). (D2O:CD3OD 4:1, 600 MHz)
Fig. S22. 1H-13C HMBC NMR spectrum of naringenin-7-O-sulfate (compound 4). (D2O:CD3OD 4:1, 600 MHz)
